# A Bibliometric Analysis of Mexican Bioinformatics: A Portrait of Actors, Structure, and Dynamics

**DOI:** 10.3390/biology11010131

**Published:** 2022-01-13

**Authors:** Dagoberto Armenta-Medina, Christian Díaz de León Castañeda, Alma Armenta-Medina, Ernesto Perez-Rueda

**Affiliations:** 1 Consejo Nacional de Ciencia y Tecnología (CONACYT), Ciudad de México 03940, Mexico; christian.diaz.de.leon@umich.mx; 2 Centro de Investigación e Innovación en Tecnologías de la Información y Comunicación (INFOTEC), Aguascalientes 20326, Mexico; 3 Universidad Michoacana de San Nicolás de Hidalgo (UMSNH), Morelia 58030, Mexico; 4 Laboratorio Nacional de Genómica para la Biodiversidad (LANGEBIO), Unidad de Genómica Avanzada, Centro de Investigación y Estudios Avanzados del IPN (CINVESTAV-IPN), Irapuato 36821, Mexico; alma.armenta@gifs.ca; 5 Global Institute for Food Security (GIFS), University of Saskatchewan, Saskatoon, SK S7N 4L8, Canada; 6 Unidad Académica de Yucatán, Instituto de Investigaciones en Matemáticas Aplicadas y en Sistemas (IIMAS), Universidad Nacional Autónoma de México (UNAM), Mérida 97302, Mexico

**Keywords:** bibliometrics, bibliographic databases, Informatics, health, Information science, bioinformatics, computational biology

## Abstract

**Simple Summary:**

This work analyzes the development of Bioinformatics research in Mexico through articles published in the last 25 years, as they have been stored in a field-specific database (PubMed). The main findings reveal that the main themes identified are strongly related to the research of outstanding researchers, the outstanding collaborations of Mexican institutions with foreign countries and institutions are influenced by the geographic proximity and binational agreements as well as philanthropic and academic programs that promote collaborations, and there is an inclination for health issues promoted by public health financing and philanthropic organizations. It is identified that publications in the field had an explosion since 2012 due to the maturity of nucleic acid sequencing technologies and the high availability of this information in public databases. Overall, this work suggests both the necessity to improve funding in this informatic discipline and promote academic collaboration inside and outside the country. Finally, the strategy used in this work allows us to make a diagnostic of the strengths and weaknesses of bioinformatics research development that can be applied in other countries. This is worth it because bioinformatics is a tool that can boost several economic sectors.

**Abstract:**

Bioinformatics is a very important informatics tool for health and biological sciences, focusing on biological data management. The objective of this work was to perform a bibliometric analysis regarding the development of Mexican bioinformatics. An exhaustive revision of the literature associated with Mexican bioinformatics in a period of 25-years was performed. Bibliometric tools, such as performance analysis and science mapping were included in the analysis. We identified the main actors as well as the structure and dynamics of Mexican bioinformatics. Some of the main findings were as follows: the thematic structure in the field is defined by the research lines of outstanding authors; the outstanding collaborations of Mexican institutions with foreign countries and institutions are influenced by the geographic proximity and binational agreements, as well as philanthropic and academic programs that promote collaborations, and there is an inclination for health issues promoted by public health financing and philanthropic organizations. It is identified that publications had an explosion since 2012, we consider that this growth may be influenced by the democratization of data, derived from the mass sequencing of biological molecules stored in public databases.

## 1. Introduction

Bioinformatics, in its minimalist definition, is the application of computational technologies to the management and analysis of biological data [1]. The term bioinformatics was coined by Paulien Hogeweg and Ben Hesper to describe “the study of informatic processes in biotic systems”. Bioinformatics is an interdisciplinary field involving many different types of specialists, including biologists, molecular biologists, computer scientists, and mathematicians [2,3].

The current omic technologies (genomics, proteomics, and metabolomics) have generated a vast amount of data without precedents in the history of biology, creating the need for their storage [4], annotation, analysis, organization and integration in biological networks and databases. These technologies have been the leading forces for the growth and development of bioinformatics in the last years [5], however, bioinformatics is not just about storing biological data, it also concerns conducting experiments on that data.

The challenge of managing the large volume of omic data is only possible thanks to the computational power, algorithms, and methods used by bioinformaticians, which help to reveal unknown aspects of biological systems [5] that have led to an explosion of publications in this area. In general, bioinformatics is considered by several countries to be one of the main areas for the innovation of technologies related to the life sciences and of high relevance for the development of the global bio-economy [6,7,8].

To reveal the state of global bioinformatics, several studies have been carried out using bibliometric tools and statistical analyses. Bibliometrics is a set of methods used to study or measure information from a text, specifically in large collections of data, and we can divide it into two main areas: Performance Analysis and Scientific Mapping. Performance Analysis helps to evaluate groups of scientific actors (countries, universities, and researchers) and the impact of their activity based on the bibliographic data. Scientific mapping focuses on monitoring and delimiting a research area to determine its thematic structure and evolution [9]. Most bibliometric studies on bioinformatics have focused on understanding the scientific production of the area by countries and the exploration of social and geographical relationships between institutions [10,11].

Since bioinformatics plays an increasingly critical role in the development of biosciences and national bioindustries, it is imperative to analyze the scientific productivity of this area. Regarding the development of bioinformatics in Latin American countries, a previous study was published from a bibliometric approach [12]. According to this study, the main themes within Latin American bioinformatics correspond to the production and analysis of genomic data, followed by proteomics studies and structural bioinformatics studies. These themes are not specific to the Latin American countries, but rather recapitulate the world growth around the new technologies of life sciences with impact in bioinformatics. Additionally, this preliminary analysis reveals that the countries with the highest production of literature in Latin American bioinformatics are Mexico and Brazil.

However, a depth study of bioinformatics development is necessary per country. Previous work explored the scientific productivity and situational diagnosis from the academic perspective in Mexico [13], but did not perform an integral analysis including productivity, conceptual structure, and thematic evolution of the research in the field. Understanding the conceptual structure of the area as well as its thematic evolution will help to reveal the dominant, transversal and emerging themes of national bioinformatics, generating a map on which strategies can be developed to exploit the most relevant themes, strengthen emerging ones and promote those relatively new concerning the international context.

The main goal of this study is to have a general perspective of the structure and dynamics of Mexican bioinformatics over a 25-year window; accurately, identify what institutions have the highest scientific production in the area, the leading players, countries, and foreign institutions that collaborate with Mexico, scientific journals, and authors with more representation that reveal clues about the current state and future of the area. Besides, the integrative approach of science mapping allows us to understand the main issues on which national bioinformatics focuses and what have been the dynamics of these over the last 25 years.

## 2. Materials and Methods

### 2.1. Bibliographic Recovery Associated with Mexican Bioinformatics

A bibliometric analysis was performed using the PubMed database [14] (the bonafide database for the biomedical literature that covers the most significant number of journals associated with bioinformatics [11]) on the scientific articles related to Mexican bioinformatics. For the bibliographic search, we used the following keywords: Mexico [affiliation] AND (bioinformatics OR ‘computational biology’ OR (computational AND genomics) OR (computational AND transcriptomics) OR (computational AND proteomics) OR bioinformatics [Journal] OR (computational AND protein) OR (computational AND DNA) OR (computational AND RNA) OR (Systems biology))”.

The Mexican bioinformatics articles were selected including those where at least one of the authors had an affiliation with a Mexican institution and were published between 1994 and 2018. This bibliography was used to evaluate the productivity, structure, and dynamics of Mexican bioinformatics using the R programming language [15], including the ggplot2 and bibliometrix libraries [16]. We measured the number of publications over the years to assess productivity, identifying the outstanding journals, Mexican and foreign institutions, and authors.

### 2.2. Country of the First Authors

For each paper selected we identified the country where the affiliation of the first author is located, then enumerated the number of first authors for each country. The number of first authors for a country could suggest a relevant contribution of the country to the literature.

### 2.3. Word Co-Occurrence Networks and Country Collaboration Network

After generating a network of co-occurrence of words contained in the titles of articles associated with Mexican bioinformatics, the stop words of the titles were eliminated to calculate the co-occurrence index of the words. The frequency of co-occurrence of two words in the titles of the articles is determined from the corpus of documents, by counting the number of documents in which two words appear together in the title. We construct a network from the 40 nodes belonging to the words extracted from the titles with the highest degree of co-occurrence according to the association strength index considered as the most appropriate metric for the normalization of co-occurrences frequencies [17,18]. In Equation (1), *C_ij_* is the count of publications in which two keywords *i* and *j* co-occur, and *C_i_* and *C_j_* represent the count of publications in which each one appears.

(1) $$e_{ij}=c_{ij}^{2}/c_{i}c_{j}$$

In colors, the word co-currency clusters are observed according to the walk trap clustering algorithm [19]. The country collaboration network was generated using ten nodes (countries with the most outstanding collaborations within Mexican bioinformatics) using the circle shape as a map layout without clustering.

### 2.4. Theme Selection

Once the abstracts of the articles associated with Mexican bioinformatics were obtained, the words contained in the title of the articles related to Mexican bioinformatics were chosen for co-word analysis [20]. The next step is the calculation of similarities based on the frequency of co-occurrences of the words in the titles, using the association strength metric for the normalization of co-occurrences frequencies [17,18].

The final step was clustering for the location of subgroups of words that are strongly linked with each other using the simple center algorithm [17]. The simple centers’ algorithm is well known in many studies in the context of word-matching [21,22] and automatically returns the clusters labeled by using different parameters, such as minimum word frequency and cut-off values for the co-occurrence, also the algorithm has two parameters to limit the size of the detected themes, the maximum and minimum sizes of the network which can be adjusted by experts in the field. We set these parameters using the 519 most abundant words of article titles for the 25 years and a minimum word cluster frequency of 5. Previous to word selection, stopwords were removed from the titles to select the most relevant terms.

### 2.5. Strategic Diagrams

Co-word analysis was used to obtain word clusters and their interconnections. These clusters are topics or themes, and each research theme obtained in this process is characterized by two parameters: Callon’s density and centrality [17].

Centrality evaluates the degree of interaction among clusters defined as Equation (2) with *k* a keyword belonging to the theme and *h* a keyword belonging to other themes. The density measure defined as Equation (3) can evaluate the internal strength of the clusters, with keywords *i* and *j* belonging to the theme, and w is the keyword count in the theme.

(2) $$c=10\times \sum e_{kh}$$

(3) $$d=100\left(\sum e_{ij}/w\right)$$

Strategic diagrams are a two-dimensional space constructed by graphing topics according to their ranking of values in density and centrality used to classify the themes into four quadrants [17,22,23].

The strategic diagrams used here were considered because they have a better interpretation of the results employing the categorization of the different themes [23]. The area of the spheres is proportional to the number of documents corresponding to each word of a theme. Each thematic network is labeled using the name of the most significant word associated with the theme (usually identified as the most central word).

### 2.6. Thematic Evolution

Raw data were divided into different groups of consecutive years or sub-periods to analyze the evolution of the field under study. Bibliometric maps of thematic evolution are constructed by joining Tt (*U* themes of a subperiod) and Tt + 1(*V* themes in the next subperiod) through “conceptual nexus” or link. A link of the *U* themes to the *V* themes is generated if there is a thematic link between them. In other words, if they have elements in common (words). The importance of the thematic link can be weighted by elements that the two themes have in common. We used the Inclusion index [21,24,25] defined in Equation (4) to perform this task.

(4) $$Inclusion\_index=\frac{\#\left(U\cap V\right)}{min\left(\#U,\;\;\;\#V\right)}$$

The Inclusion index has the advantage of being more useful for the measurement of similar sets compared to other indices, such as the Jaccard or the cosine since it is not biased by the number of items objects like the latter two [24]. The Inclusion index will be equal to 1 if the words of theme *V* are completely contained in theme *U*. For this reason, and since the weight of the thematic nexus is a good measure of overlapping between themes, the Inclusion index has been chosen as in previous works [9,25,26].

It is important to note that depending on the interconnections between them, the same theme may belong to different thematic areas, or it may go to none. Additionally, themes with the same name can be maintained through the periods within a thematic area, or a thematic area can be composed of themes with different names. The thickness of the arrows is proportional to the Inclusion index, and the area of the boxes is proportional to the number of published articles associated with each theme.

The three periods used to analyze the thematic evolution of the area include the first period from 1994 to 2006 with 121 publications, the second period from 2007 to 2012 with 268 publications, and the last period from 2013 to 2018 with 1453 publications. For the last two periods, we considered periods of 6 years, unlike the first period that was extended to 12 years (due to the low number of articles) to achieve greater robustness in the analysis. The methodology used to obtain the thematic map and the analysis of thematic evolution is based on the network of co-word analysis developed by Cobo [9], we use the 110 most abundant words of article titles for each period and a minimum word cluster frequency of 5.

## 3. Results

### 3.1. Publication Dynamics

**Figure 1:**
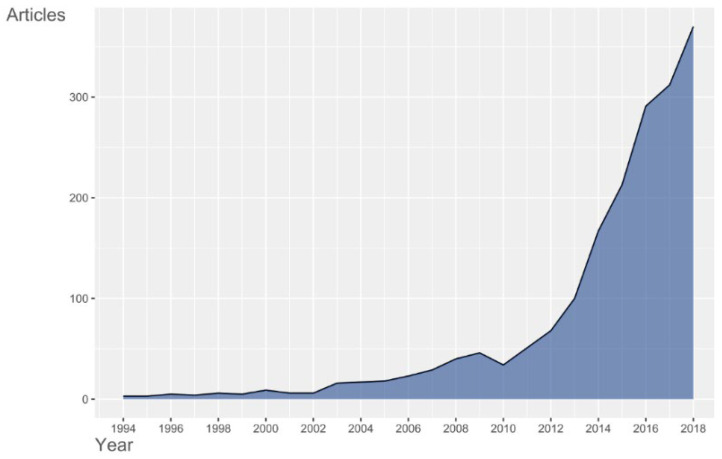
Dynamics of publications per year associated with Mexican bioinformatics. In *X*-axis is indicated the year for a window of 25 years. *Y*-axis is indicated the number of articles published.

We obtained a total of 1842 publications associated with Mexican bioinformatics from the PubMed database. The dynamics of scientific production associated with Mexican bioinformatics range from low production in the 1990s with a slight increase in 2004 to an explosion starting in 2012 (Figure 1). The beginnings of bioinformatics in Mexico date back to the 1990s with few articles published. In 2004, possibly derived from the implementation of various molecular biology techniques and the excitement of the human genome sequencing project, a slight increase in the number of publications is observed. In 2012, the maturity of mass nucleic acid sequencing technologies and the high availability of this information in public databases could favor the explosion in the number of publications associated with Mexican bioinformatics.

### 3.2. Most Relevant Journals Associated with Mexican Bioinformatics

We found that around 39 journals make up the core journals of articles associated with Mexican bioinformatics (Table 1, and the complete list in Table S1). The heterogeneity of the journals associated with the national bioinformatics goes from multidisciplinary journals, such as *PLoS ONE*, *Proceedings of the National Academy of Sciences of the United States of America* (*Proc. Natl. Acad. Sci. USA*), and *Scientific Reports* (*Sci. Rep.*) to more specialized journals, such as *BMC Genomics*, *Journal of Proteomics*, and *Bioinformatics*. Additionally, as expected, we observed national magazines, such as the *Gaceta Medica de Mexico* (*Gac Med Mex*) and *Salud Publica de Mexico* (*Salud Publica Mex*), where bioinformatics analysis is published. The fact that both are journals of national origin favors their visibility within Mexican researchers, interestingly, most of these journals are associated with the health sector.

### 3.3. Institutions with a More Significant Presence in the Production of Literature Associated with Mexican Bioinformatics

The analysis performed allowed us to identify the most outstanding academic and research centers working in bioinformatics in Mexico. As expected, the two largest research institutions in Mexico: Universidad Nacional Autónoma de México [National Autonomous University of Mexico] (UNAM) and the Instituto Politécnico Nacional [National Polytechnic Institute] (IPN) are those with the highest scientific output associated with bioinformatics. Within the UNAM, the Center of Genomic Sciences (CCG-UNAM) and the Institute of Biotechnology (IBT-UNAM) stand out. Within the IPN, the Centro de Investigación y Estudios Avanzados [Center for Research and Advanced Studies] (CINVESTAV), the Laboratorio Nacional de Genómica para la Diversidad [National Laboratory of Genomics for the Biodiversity] (LANGEBIO-CINVESTAV), and the Escuela Superior de Medicina [Higher School of Medicine] (ESM-IPN) stand out.

Other very important institutions for Mexican bioinformatics are part of the health system, such as the Instituto Nacional de Medicina Genómica [National Institute of Genomic Medicine] (INMEGEN), and the Instituto Nacional de Salud Pública [National Institute of Public Health] (INSP), both pertaining to the Institutos Nacionales de Salud, Secretaría de Salud [National Health Institutes, Ministry of Health]. Additionally, the Instituto Mexicano del Seguro Social [Mexican Institute of Social Security] (IMSS), pertaining to the social security schemes of the health system. These health institutions stand out in bioinformatic publications related to diverse health problems like cancer and diabetes.

Regarding foreign institutions located in Mexico, it is worth mentioning the International Maize and Wheat Improvement Center (CIMMYT) focusing on topics of great interest, such as the cultivation of corn, food for essential consumption in the country. The foreign institutions located outside Mexico with the most relevant collaboration associated with Mexican bioinformatics are from the USA, with a focus principally on the health sector, such as Baylor College of Medicine, Broad Institute of MIT and Harvard, Harvard Medical School, and the University of California, which occupy the first place. Interestingly foreign institutions with the most relevant collaboration suggest that geographical proximity could be a factor that favors binational scientific interaction (Table 2).

### 3.4. Outstanding Authors in the Number of Publications

In the list of relevant authors associated with Mexican bioinformatics, the bioinformatician Dr. Collado-Vides, emerge as one of the pioneers of Mexican bioinformatics, who has maintained a line of research on the regulation of gene expression at the CCG-UNAM followed by Dr. Correa-Basurto and Dr. Bello from IPN (Table 3).

### 3.5. Country of the First Authors

**Figure 2:**
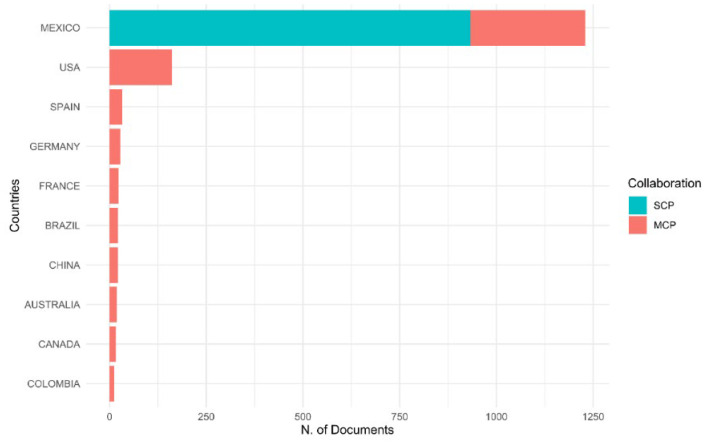
The number of articles associated with the different countries related to Mexican bioinformatics. Only the ten most outstanding countries related to Mexican bioinformatics are plotted. The label SCP refers to single publication country publications, and MCP corresponds to multiple publications countries.

The articles selected as part of Mexican bioinformatics reflect an essential contribution of the Mexican scientific activity associated with bioinformatics. Mexico is ranked first in the number of publications with first authors associated with a Mexican research institution, which mostly correspond to publications of a single country (Single Country Publication-SCP) (Figure 2). On the other hand, as expected, all articles with first authors not belonging to Mexican organizations correspond to publications from multiple countries (Multiple Country Publication-MCP), which represent less than half of all publications. Additionally, the United States of America occupies second place with first authors, which suggests that factors, such as geographical proximity favors interaction with Mexican scientists in this area of research.

### 3.6. Main Countries and Collaborating Institutions Associated with Mexican Bioinformatics

**Figure 3:**
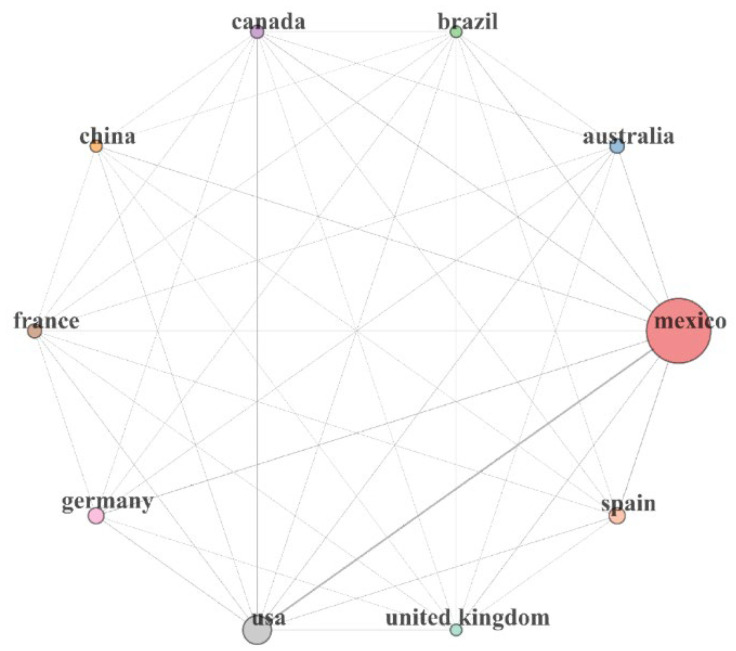
The network of countries collaborating with Mexican bioinformaticians. The area of the circle reflects the proportion of articles where each country collaborates in the literature associated with Mexican bioinformatics.

The countries that stand out as principal collaborators of Mexican bioinformaticians are the United States of America due to the leadership of this country in the bioinformatics area and its geographical proximity, among other factors discussed in the next sections. The shared language and cultural influence could explain collaboration with other countries, such as Spain; other collaborations are with leading countries, such as Germany and France (Figure 3).

### 3.7. Word Co-Occurrence Networks Analysis

**Figure 4:**
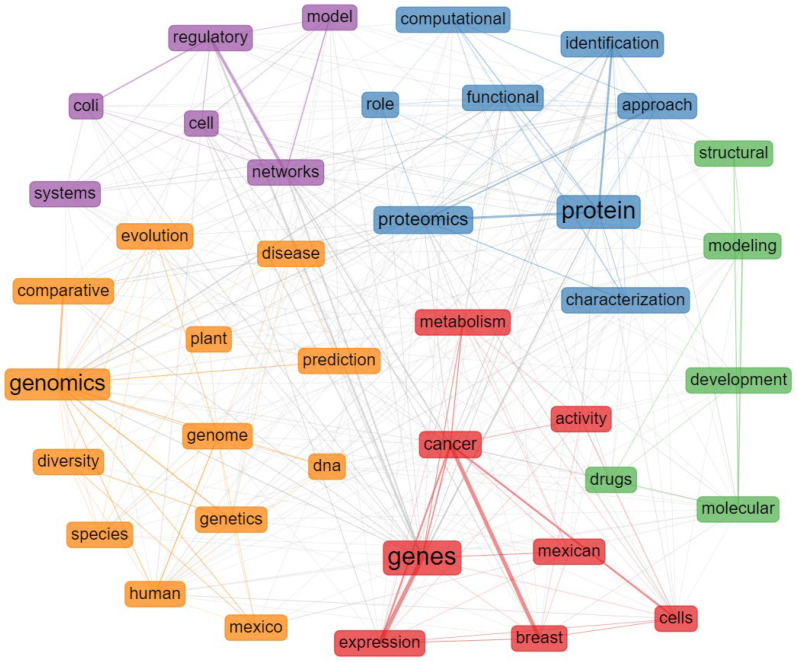
Word co-occurrence network extracted from the titles of the articles associated with Mexican bioinformatics. Clusters in colors are derived from the walk trap algorithm. The word size is proportional to occurrences and the edges to the association strength index between words.

The network of co-occurrence of words belonging to the titles of the publications reflects four main clusters that define the structure of the field (Figure 4). The purple cluster was associated with *Escherichia coli* and bacterial regulatory networks, which is coherent with the research topic of one of the pioneers of bioinformatics in Mexico, Dr. Collado-Vides. The green cluster contains terms associated with protein analysis, such as “molecular”, “modeling”, “drug” and “structural” related to the research area led by Dr. Correa-Basurto, also one of the outstanding authors in our study. In yellow, we found another cluster related to evolutionary and comparative genomic analysis of species. The cluster in red is observed to be more related to the study of diseases, such as cancer through analysis, such as gene expression in the Mexican population closely related to the lines of research developed by Dr. Hernandez-Lemus (INMEGEN).

### 3.8. Thematic Map of Mexican Bioinformatics

**Figure 5:**
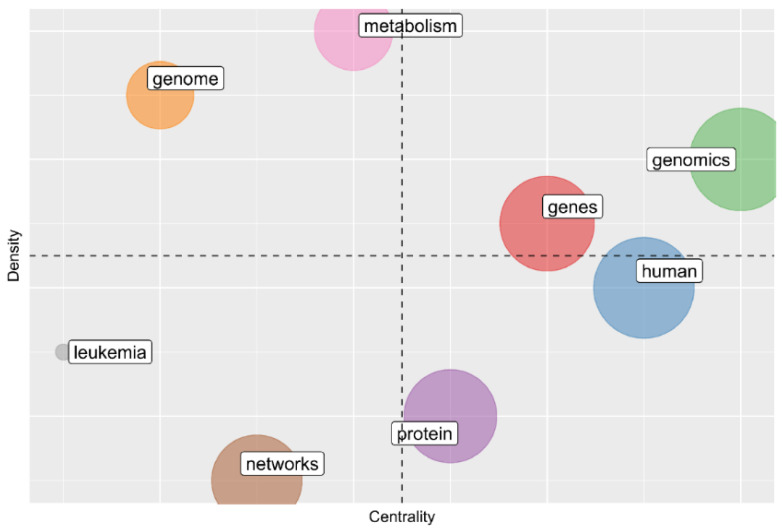
Thematic map of Mexican bioinformatics corresponding to the 1994–2018 period. The circle area represents the abundance of a theme in the literature. The *X*-axis represents Callon’s centrality of the themes in the network, and the Y-axes the density.

We found four types of themes according to the quadrant in which they are located [17,23](Figure 5). Themes in the upper right quadrant are those that are well developed and are essential for the structure of the research field and are externally related to concepts applicable to other themes. Within this quadrant, there are three main themes labeled with the words “genes” and “genomics.” These are known as motor themes since they present a strong centrality and high density in the network of co-occurrence of words. Themes in the upper left quadrant maintain well-developed internal but not external relationships. These themes are very specialized and peripheral, highlighting the themes “genome” and “metabolism” (Figure 5).

Themes in the lower left quadrant are considered marginal and underdeveloped due to their low density and centrality in the network and could represent emergent or disappearing themes. The theme “leukemia” is associated with the bioinformatics study of lymphoblastic cancer in Mexican children; another highly occurring concept associated with the theme is the word “lymphoblastic”. The “networks” theme is associated with the use of bioinformatics tools for the understanding of gene regulation phenomena through cellular interaction networks (Figure 5).

Themes in the lower right quadrant group are basics general and transversal themes. The subject of “protein” is in this quadrant, and also “human” which indicates that these themes are important for the research field, however, they are not fully developed in Mexico (Figure 5).

### 3.9. Thematic Evolution Analysis

Co-word analysis techniques can help to evaluate the evolution of a field of research through a longitudinal study, allowing us to reveal the structure and dynamics of research themes [27,28]. In this regard, the thematic evolution determined for the period 1994–2018 was divided into three sub-periods, the first one that goes from 1994–2006, the second period that goes from 2007–2012, and the third period that goes from 2013–2018. Although it is desirable to use the same years for each period, the first years of scientific production associated with Mexican bioinformatics were scarce, so we lengthened the period to have several essential terms.

Therefore, there are different degrees of continuity that can be measured with several indexes of similarity. In this sense, the Inclusion index has been used to measure continuity between clusters due to the advantages mentioned in previous works [27,29].

**Figure 6:**
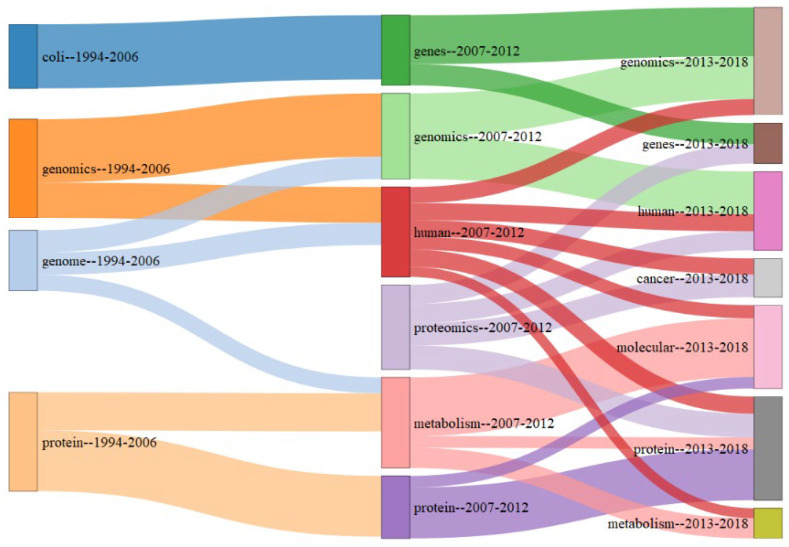
Thematic evolution map of Mexican bioinformatics over a 25-year window divided into three periods. The thickness of the arrows is proportional to the Inclusion index. The box area is proportional to the abundance of the theme in the literature.

In Figure 6, themes of the first and second period are connected, which suggests that most of the themes of the second stage are linked and emerged due to the influence of themes of the first stage where the “genome” theme, shows a high degree of reticulation, as shown by its size in the blue lightbox on Figure 6. We also found a growth in the number of themes from the first period to the second period, going from four to six themes probably due to the increase in the number of publications in the field, generating new themes like “human” and “proteomics”. The themes conserved between the first and second periods are “genomics” and “protein”, the first theme starts with the study of complete sequenced genome of model organisms and is widely studied with the increase of sequenced genomes, while the “protein” theme has also been one of the most widely studied through bioinformatics tools to understand its function and structure.

From the second period to the third period, we did not see any relevant change in the number of themes, i.e., we observed conservation of the “genomics” and “human” themes. In the third period, the new “cancer” and “molecular” themes stand out. In general, in the third period, new labeled themes are observed with concepts that involve a study of greater complexity and data integration, such as the “cancer” and “molecular”.

## 4. Discussion

This section provides some additional notes regarding the two main approaches used in this study: (1) performance analysis, which allows detecting the main actors associated with Mexican bioinformatics and its influence, and (2) science mapping, which allows analyzing the conceptual structure to understand the achievements and evolution of the area through time.

### 4.1. Performance Analysis

#### 4.1.1. Number of Publications per Year

The first years have a low number of publications, with an explosion of scientific literature associated with Mexican bioinformatics, posterior to 2012. The knowledge derived from bioinformatics studies has increased rapidly since the 1990s at the international level and is expected to grow, favored by high-performance experimental technologies [30,31]. In this regard, Patra and Mishra [31] observed an exponential growth between 1996 and 2000, which they attribute to the explosion in genomic research followed by the resolution of the human genome project. In this sense, it is evident that Mexican bioinformatics has a delay of more than ten years in the number of publications regarding international bioinformatics. This phenomenon may be because Mexico is not an innovator in high-performance experimental technologies, such as the omics and depends strongly on the adoption and assimilation from other countries.

**Figure 7:**
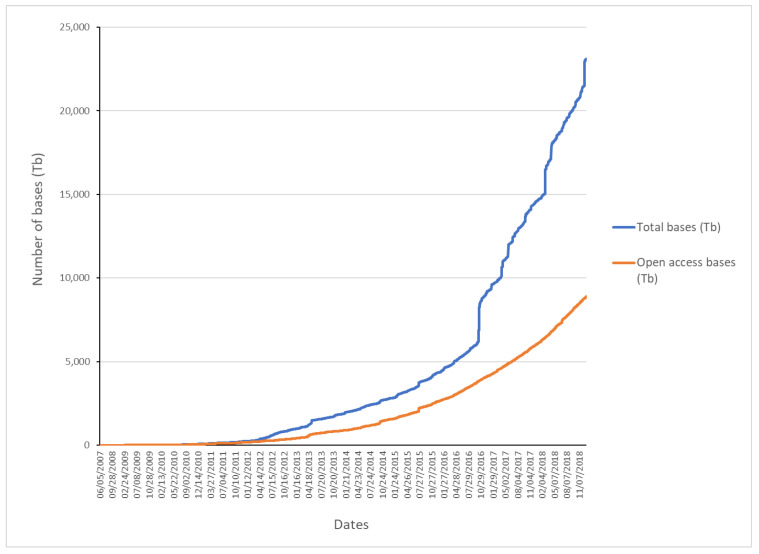
Evolution of the SRA, which stores raw data from massive sequencing projects. The total number of bases in Terabytes (Tb) in blue color and of exclusively public access in red color.

A phenomenon that may be associated with the growth in the number of publications associated with Mexican bioinformatics is the availability of biological information in public databases, the democratization of this information facilitates the use of bioinformatics analysis without the need to have direct access to an extensive infrastructure or resources to finance research in bioinformatics. Finally, the explosion in the number of publications from 2012 could also be associated with the maturation of massive sequencing technologies, this suggestion is supported by the increase in the growth of public databases of raw sequences, such as the Sequence Read Archive database (SRA) [32] (Figure 7) where a considerable increase around 2012 is observed. The availability of these public databases favors various bioinformatics analyzes that eventually result in new publications.

#### 4.1.2. Main Journals

The most prominent journals in the number of publications associated with Mexican bioinformatics are generalists, such as *PLoS ONE*, *PNAS*, and *Scientific Reports*. The fact that most of the journals are generalist denotes the multidisciplinary approach of Mexican bioinformatics and its use as a complementary tool to the different biological science research areas, rather than as a developer of new methodological approaches, such as those found in specialized journals. However, to a lesser extent, we also observe journals specialized in bioinformatics, such as *Bioinformatics* and *PloS Computational Biology*, which suggests that there are isolated efforts in the development of methods and databases. Many of the leading journals in Mexican bioinformatics are also found in the core of journals associated with worldwide bioinformatics [11,31], although interestingly, *PLoS ONE* and *Scientific Reports* do not appear in the core of journals associated with worldwide bioinformatics because the journals mentioned above are relatively new.

#### 4.1.3. Main Institutions

The ANUIES (Asociación Nacional de Universidades e Instituciones de Educación Superior [National Association of Universities and Institutions of Higher Education]) reports 207 public and private higher education institutions in Mexico [33]. Interestingly, within the institutions with the greatest collaboration in Mexican bioinformatics included in the top 15 (Table 2), there are seven Mexican public institutions, including the two main higher education institutions (UNAM and IPN) and health institutions (INMEGEN, INSP, HIMFG and IMSS). However, there is an important gap in academic production between the first two Mexican institutions (UNAM and IPN) and the subsequent ones, which may be because they cover a large number of topics related to bioinformatics, unlike the institutions that specialize only in research in this field.

Comparing the UNAM and IPN with the other institutions of higher education in the country, which do not appear in Table 2 (except the UAG), these institutions have a greater history and infrastructure, and possibly more research funding. In addition, it is worth mentioning that they are mainly centralized in Mexico City, which is the capital of the country, showing the gap in academic and research development that exists in the interior of the country.

Among the international institutions with the most significant collaboration with Mexican bioinformaticians is the University of California, which is not a surprise since previous studies point to it as the most outstanding global institution in bioinformatics publications [31]. Other factors that favor collaboration are its geographical proximity and bilateral agreements, such as the UC-MEXUS program [34]. UC-MEXUS promotes the exchange of researchers from various areas between Mexico and the United States of America, favoring academic interaction, which reflects in publications showing the success of these binational policies.

Other outstanding international institutions in collaboration with Mexico are the Baylor College of Medicine, the Broad Institute of MIT and Harvard, Harvard Medical School, all these institutions located in the United States. Besides geographical proximity, other factors that promote collaboration are scholarship support programs by the Mexican government, specifically Consejo Nacional de Ciencia y Tecnología (CONACYT) study grants and foundations, such as the Comision México Estados Unidos para el Intercambio Educativo y Cultural [US-Mexico Commission for Educational and Cultural Exchange] (COMEXUS) with binational support programs [34].

Within the private and philanthropic sector, the Fundación Carlos Slim [Carlos Slim Foundation] (FCS) stands out interestingly since it has financed collaborative projects between some USA and Mexican institutions. For example, the FCS favored the collaboration of the Baylor College of Medicine with the Universidad Autónoma de Yucatán [Yucatan Autonomous University](UADY) and the CINVESTAV, financing a project of about 2.6 million dollars for the development of a vaccine against Chagas disease [35,36]. In addition, to understand the genomic bases of cancer and type II diabetes in Latin American and Mexican populations, the FCS through an initiative for genomic medicine [37] financed a project with around 134 million dollars in two stages, promoting collaboration between the Broad Institute of MIT and Harvard, the Instituto Carlos Slim de la Salud [Carlos Slim Health Institute] and the INMEGEN.

Additionally, in the case of Harvard, there is support from the Mexico Foundation at Harvard (FMH) for masters and doctorate studies under the agreement that involves the Harvard/CONACYT/FMH institutions. All the above suggests that the philanthropic sector has been favoring binational collaborations in bioinformatics associated with health.

#### 4.1.4. Main Actors

The outstanding authors of Mexican bioinformatics come mainly from the two largest academic institutions, UNAM and IPN. The list is headed by the pioneer bioinformatician Dr. Collado-Vides (CCG-UNAM) and other former members of his research group sharing publications in the area of regulation of gene expression in bacteria stand out also. Dr. Correa-Basurto and Dr. Bello (IPN) share the area of structural biology focusing on drug design. Dr. Treviño (Instituto Tecnológico de Monterrey [Technological Institute of Monterrey], ITESM) focuses on the development of bioinformatics tools for health issues. Dr. Merino (IBT-UNAM) stands out with the study of regulatory mechanisms in bacteria and Dr. Sanchez-Flores in the area of genomics. Dr. Hernandez-Lemus (INMEGEN) stands out with lines of research in computational and population genomics in various topics, such as cancer.

Regarding the bibliometric technique used in this section, it should be noted that the detection of relevant authors with other indicators, such as the H index does not result in substantial differences for the top authors based on the number of articles, although they do include new leaders, such as Dr. Abreu, who directs a computational RNA genomics group at LANGEBIO-CINVESTAV.

#### 4.1.5. Main Countries Linked to Publications Associated with Mexican Bioinformatics

Among the main collaborating countries with Mexico, the United States emerged as the principal, followed by Spain, Germany, Australia, France, and Brazil. Previous bibliometric studies reveal the United States, Germany, France, and to a lesser extent, Australia and Spain as leaders of global bioinformatics [11,31]. The leadership of the United States in the area, its geographic proximity, and exchange of researchers favor interactions, which is reflected in the number of publications. Spain also stands out as a collaborator who can be explained by questions of cultural and idiomatic relationships. Brazil is one of the fastest-growing emerging countries in the area of bioinformatics [12], which also favors collaborations in publications associated with Mexican bioinformatics.

### 4.2. Science Mapping

#### 4.2.1. Co-Occurrence Network

Analyzing Figure 4, the cluster in purple reflects the lines of research in regulation of the expression of the research group of Dr. Collado-Vides and his disciples. The red cluster with words, such as “cancer”, “metabolism”, “expression”, and “Mexican” is closely related to the research lines of the INMEGEN and Dr. Treviño (ITESM), as mentioned in the previous section the study of cancer and metabolic diseases in the Mexican population has been favored by binational research support through philanthropic financing [37,38,39,40]. The yellow cluster highlights the word genomics, reflecting all the research trends that have been influenced by the massive sequencing technologies where Dr. Sanchez-Flores (IBT-UNAM) stands out. As for the green cluster, its concepts are associated with the area of structural biology and drug design, one of the areas with most tradition in international bioinformatics, where outstanding authors, such as Dr. Correa-Basurto and Dr. Bello (IPN) stand out. In general, the clusters of the co-occurrence network derived from the words of the titles of the publications associated with Mexican bioinformatics reveal that the thematic structure of the area has taken shape and has been influenced by the research lines of the principal authors of Mexican bioinformatics and also by public and philanthropic funding agendas.

#### 4.2.2. Strategic Diagram

In the upper right quadrant of Figure 5 associated with the motor themes of Mexican bioinformatics within the theme labeled as “genes”, the concepts of higher occurrence related to this theme are “cancer”, “expression” and “breast”. All these concepts reflect the study of the molecular basis of cancer in the Mexican population driven by the funding of the public and philanthropic sectors [35,37,41,42]. The “genomics” theme also belonging to this quadrant contains the concepts of high occurrence as “genomics”, “genetics”, “mexico”, “comparative” and “data”, this theme highlights concepts associated with massive nucleic acid sequencing technologies which have generated a large amount of data that has made possible the comparison on a large scale of the different biological species and populations in Mexico using bioinformatics tools [4].

In the left upper quadrant of Figure 5 are the themes of high specialty in the field with a considerable density but not sufficiently central as the motor themes. One of the themes that stand out in this quadrant is the “genome” theme, which contains the higher occurrence concepts like “effects”, “common”, “sequence”, “strain”, “isolated” suggesting the relation of this theme with the genome identification and characterization of species and ecotypes. The “metabolism” theme contains the concepts “disease”, “type” and “diabetes” which reveals the specialized study one of the biggest public health problems in Mexico [42]. The “metabolism” theme also contains a concept of higher occurrence “metabolomics” related to the use of bioinformatics tools for the characterization and identification of metabolites derived from massive technologies [43].

The lower left quadrant of Figure 5 has themes of low centrality and density that can represent emerging or disappearing themes. Within the theme “leukemia” we found the high occurrence concepts like “lymphoblastic”, which implies that it could be an emergent theme of specialization in the study of leukemia currently known as one of the biggest health problems in Mexican children [44,45]. Another theme that stands out is “networks”, where high occurrence concepts, such as “regulatory”, “coli” suggests that this theme has been influenced by one of the pioneering topics (gene regulation) of Mexican bioinformatics led by Dr. Collado-Vides and additional terms like “system”, “model” suggest an integrative stage of the theme for the study of gene regulation. This theme is close to an intermediate centrality suggesting their expansion through the accumulation of data derived from the new technologies of massive sequencing contributing to their growth and maturation. At the lower right quadrant of Figure 5, highly central themes with incomplete development are located; these are considered general or basic themes and in the case of Mexican bioinformatics, include “human” and “protein” themes. The human theme contains the concepts “molecular”, “DNA”, “drugs”, “modeling” and “computational” all these related to structural biology for drug discovery. The human theme is also related to the research focus of Dr. Correa-Basurto, one of the tops authors related to Mexican bioinformatics. The outstanding concepts of “proteomics”, “characterization”, “plant” and “species” belong to the protein theme and reflect the extensive characterization and identification of protein molecules in different species through bioinformatics and new sequence technologies [43]. The low development is associated with a low cohesion between the internal concepts of these themes that happens when they are broad and ambitious.

Unlike international literature on worldwide bioinformatics [11], there is no outstanding occurrence of terms, such as “algorithms”, “databases”, “software” associated with the Mexican bioinformatics (See Table S2) which denotes that Mexican bioinformatics is still mainly using bioinformatics software applications developed in other countries and with few efforts focused on the development of new applications. The bioinformatics application development could be a new thematic area to build and promote in Mexican bioinformatics.

#### 4.2.3. Thematic Evolution

In the first stage of Figure 6, we found “coli” and “genome” themes, this is explained because *E. coli* was one of the first model organisms with a complete genome, widely used for characterization studies through molecular biology [46]. At this stage, many of the bioinformatics were focused on the comparative study of sequences and pattern search in this organism and other genome models [47].

Another relevant theme in the first period is “protein” related to characterization studies and function assignment of molecular components where bioinformatics tools were used mostly for gene prediction and function assignment, at this stage, structural biology was also aided for the 3D visualization and calculation of properties associated with the structure of proteins. We could say that for this period, bioinformatics served as a support tool in reductionist studies on the characterization of molecules [48], mainly in the assignment of functions, gene search, and protein visualization, among others.

The first, second, and third periods include the theme associated with massive studies, such as “genomics” conforming to a thematic area. The growth of sequencing technologies favored the study of genomics in Mexico through public and private projects that facilitated the availability of different genome models [32].

Additionally, in the second period the theme “proteomics” emerge which due to its methodological complexity has taken longer to appear than “genomics”, the other theme that appears is “human” that contains the concepts “systems” which denotes a transition from reductionist approaches to more integrative approaches. It is essential to note the continuity and high degree of reticulation of the human theme in the third period, probably due that the human genome, being an extensive model of study, favor the development of new themes in the third period. Additionally, another emergent theme for the second period is “genes” which includes the outstanding term “networks” which implies the emergence of network analysis helped by greater availability of information and data from the reductionist approaches [49]. There is also an emergence in the third period of the “cancer” theme, as discussed earlier, probably favored by philanthropic initiatives where the complexity of the problem involves the extensive use of bioinformatics tools.

The thematic evolution allows us to see the Mexican bioinformatics focused mainly on applications of characterization and annotation of genes and genomes in the first period to more sophisticated analyses favored by the accumulation of data in the biological sciences which allow making models to greater detail favoring the development of systems biology and network analysis.

## 5. Limitations

Since bonafide databases in bioinformatics (PubMed) are English speaking mainly, abstract publications in this language were considered for bibliometric analysis only. Also, publications in less standardized databases were not included, although these probably have less relevance to Mexican bioinformatics. Another important problem faced was the variation in the names of the main actors, such as authors, organizations (universities or institutes) and countries, which was minimized using a dictionary of these variants.

For example, the names of some authors change during their careers, one or two surnames as well as one, two, or more names could be included in the articles. Due to the fact that the number of authors associated with Mexican bioinformatics is not large, it was possible to manually assign the articles to the same author by means of their adscriptions and research topics.

## 6. Conclusions

Through the bibliometric analysis of the scientific literature, we revealed aspects associated with Mexican bioinformatics, such as the main actors and collaborations of foreign countries. We detected individual geographic and cultural relationships that could be favoring the collaboration of Mexican bioinformatics with other countries and specifically binational agreements that promote the interaction of researchers with foreign Universities as in the case between the University of California and Mexican academic institutions. We also observed that the research lines of the main actors in Mexican bioinformatics influence the thematic structure of the area. We found that the most prominent themes in Mexican bioinformatics have been the study of the expression of gene regulation, structure, and function of proteins, genomics, and health issues, specifically the study of metabolic diseases and cancer promoted by public and philanthropic financing.

The study of the thematic evolution of the area helps us to understand the transition from reductionist studies, such as the characterization of genes and proteins to more integrative studies through a systems biology and network analysis for the study of more complex diseases like cancer. The comparison with studies from other countries helped us to see that Mexican bioinformatics is behind in aspects like the development of software and algorithms. The identification of the main actors of Mexican bioinformatics will serve as a starting point through surveys or interviews to identify the strengths and weaknesses of Mexican bioinformatics. Finally, the strategy used in this work, and the data revealed, could be used to understand the context of bioinformatics in other countries and give clues about aspects that favor its development.

## Figures and Tables

**Table 1:** Most relevant journals associated with Mexican bioinformatics (1994 to 2018).

Journal	Publications
*PLoS ONE*	89
*Sci. Rep.*	33
*BMC Genomics*	30
*J. Proteomics*	29
*Proc. Natl. Acad. Sci. USA*	25
*Gac. Med. Mex*	23
*Bioinformatics*	21
*Methods Mol. Biol.*	21
*Nucleic Acids Res.*	21
*Front. Physiol.*	19
*G3 (Bethesda)*	17
*Biomed. Res. Int.*	14
*Molecules*	14
*PLoS Comput. Biol.*	14
*Nature*	13
*Bol. Med. Hosp. Infant. Mex.*	12
*Int. J. Mol. Sci.*	12
*Nat. Commun.*	12
*Salud Publica Mex.*	12
*Comput. Math. Methods Med.*	11

**Table 2:** Most relevant Institutions publishing articles associated with Mexican bioinformatics for the 1994–2018 period ^a^.

Affiliations	Participation Number
Universidad Nacional Autónoma de México (UNAM)	1326
Instituto Politécnico Nacional (IPN)	553
University of California	284
Instituto Nacional de Medicina Genómica (INMEGEN)	274
Broad Institute of MIT and Harvard	246
Harvard Medical School	141
International Maize and Wheat Improvement Center (CIMMYT)	114
Baylor College of Medicine	111
Instituto Nacional de Salud Pública (INSP)	111
Universidad de Guanajuato (UAG)	104
Hospital Infantil de México Federico Gómez (HIMFG)	99
University of Copenhagen	97
Instituto Mexicano del Seguro Social (IMSS)	96
University of Cambridge	89
University of Oxford	88

^a^ The relevance is measured according to the occurrence number of an affiliation (of all co-authors for each paper).

**Table 3:** Most relevant authors by publications (1994 to 2018).

Author	Publications
Collado-Vides, J.	56
Correa-Basurto, J.	50
Bello, M.	36
Treviño, V.	34
Hernández-Lemus, E.	33
Sanchez-Flores, A.	25
Medina-Franco, J.L.	24
Salgado, H.	24
Merino, E.	21
Gama-Castro, S.	20

## Data Availability

All the information was taken from PubMed, which is a public database.

## Supplementary Materials

The following are available online https://www.mdpi.com/article/10.3390/biology11010131/s1, Table S1: Core journals of the Mexican Bioinformatics, Table S2: Word occurrence extracted from the titles of the articles related to Mexican bioinformatics (period 1994–2018).
